# Exploring the Philosophy of Mind and Its Implications for Psychiatry

**DOI:** 10.1192/j.eurpsy.2024.577

**Published:** 2024-08-27

**Authors:** M. Gomes, A. Fernandes, P. Veloso, V. Santos

**Affiliations:** ^1^Psychiatry; ^2^Hospital de Braga, Braga; ^3^Psychiatry, Centro Hospitalar Universitário Cova da Beira, Covilhã, Portugal

## Abstract

**Introduction:**

Philosophy of mind grapples with fundamental questions concerning the Consciousness, the Mind-body problem, the Identity, and Free will (as opposed to Determinism). In the context of psychiatry, this philosophical groundwork provides a conceptual framework for comprehending the intricate workings of the human psyche.

**Objectives:**

We aim to discuss how the philosophical investigation of the mind influence and enhance psychiatrists understanding of psychiatric disorders and patient-centered care.

**Methods:**

Review of the literature.

**Results:**

Philosophy of mind explores what it means to be conscious and the nature of subjective experience. This includes questions about the “hard problem” of consciousness, that refers to the difficulty of explaining why and how physical processes in the brain give rise to subjective, first-person experiences (or qualia). The “hard problem” posits that even if we knew everything about the brain’s physical processes and how they relate to cognitive functions, we would still lack an explanation for why these processes give rise to subjective consciousness. Psychiatry often deals with individuals who experience disturbances in their subjective conscious experiences, so the “hard problem” perspective allows psychiatrists to appreciate the diversity of conscious experiences and to empathize with their patients’ unique mental worlds.

Related with the previous topic is the mind-body problem. The elucidation of this problem highlights the challenge of reconciling mental phenomena with neurobiological processes. Integrating philosophical notions of dualism, materialism, and emergentism into psychiatric practice is essential for addressing the holistic nature of mental health.

Concerning to philosophical perspectives on personal identity, questions about the continuity of identity, selfhood, and the role of narrative in shaping one’s sense of self contribute to a deeper understanding of disorders like dissociative identity disorder, borderline personality disorder and even psychosis.

Furthermore, philosophical discussions on free will and determinism are pertinent to psychiatric ethics and the treatment of individuals with behavioral disorders, informing the ethical considerations surrounding involuntary psychiatric hospitalization, medication administration, and the delicate balance between autonomy and paternalism in psychiatric care.

**Conclusions:**

Philosophy of mind provides psychiatry with a rich conceptual landscape, offering insights into the nature of mental phenomena. As our understanding of the brain and consciousness continues to evolve, the philosophy of mind remains an evolving area of philosophical inquiry.

**Disclosure of Interest:**

None Declared

